# Carotid intima–media thickness is a predictor of coronary artery disease in South African black patients

**Published:** 2009-08

**Authors:** Zaiboonnisa Holland, Lucas M Ntyintyane, Frederick J Raal, Geoffrey V Gill

**Affiliations:** Department of Radiology, University of the Witwatersrand, Johannesburg, South Africa; Carbohydrate and Lipid Metabolism Research Unit, Department of Medicine, University of the Witwatersrand, Johannesburg, South Africa; Carbohydrate and Lipid Metabolism Research Unit, Department of Medicine, University of the Witwatersrand, Johannesburg, South Africa; Liverpool School of Tropical Medicine, Liverpool, United Kingdom

## Abstract

**Background:**

Several studies have shown that increased carotid intima–media thickness (CIMT) confers risk of future coronary artery disease (CAD) and stroke. The present study aimed at investigating whether CIMT is a predictor of CAD in South African black patients.

**Methods and Results:**

This was a prospective study of 53 patients, 41 men and 12 women, with ages ranging from 30 to 70 years. All patients had undergone coronary angiography for suspected CAD. B-mode ultrasound measurement of the carotid intima–media thickness was carried out in all patients, the operator being blinded to the coronary angiography findings. Twenty-nine of the 38 (76%) subjects with established CAD had increased CIMT, with an average mean CIMT of 1.13 mm. Single-vessel disease was present in 12 people, double-vessel disease in 11 and triple-vessel disease in 12. There was a significant positive linear trend between CIMT and the number of involved coronary vessels (*p* < 0.0001, *r* = 0.44).

**Conclusions:**

Increased CIMT correlated with evidence of angiographically proven CAD. The median percentile scores showed a progressive increase as the number of vessels involved increased. CIMT could be useful as a screening tool for the presence of CAD in the South African black population.

## Summary

Urbanisation, industrialisation and socio-economic development have resulted in significant changes in lifestyles globally, particularly in developing countries.^1^ An epidemiological transition is now occurring in the developing world where the major causes of death are changing from infections to non-communicable diseases of lifestyle, resulting in an increased prevalence of cardiovascular diseases such as coronary heart disease.^2^

The South African National Burden of Disease study for the year 2000 estimated that 17% of all deaths were due to cardiovascular diseases.^3^ Early detection of coronary artery disease (CAD) may well prove to be instrumental in introducing effective treatment and may contribute to reducing mortality.^4^ Thickening of the intima–media is commonly recognised as the initial stage in the development of atherosclerosis.^5^,^6^ Several studies have shown that increased carotid intima–media thickness (CIMT) confers risk of future coronary heart disease and stroke.^7^ The present study aimed at investigating whether CIMT is a predictor of CAD in South African black patients.

## Methods

This was a prospective study of 53 patients, 41 men and 12 women, with ages ranging from 30 to 70 years. All patients had undergone coronary angiography for suspected CAD.

B-mode ultrasound measurement of the carotid intima–media thickness was carried out on all patients as a blind study, the operator being unaware of the coronary angiography findings. A standardised ultrasound technique was employed, using a Toshiba System: Nemio Model SSA-550A. The transducer frequency was set at 11 MHz for all patients.

Measurement of the intima–media thickness was carried out at the optimum angle of interrogation (OAI), which allowed visualisation of the flow tip divider, the common carotid artery (CCA), external carotid artery (ECA) and internal carotid artery (ICA) from a single selected angle of the carotid arteries at the bifurcation. Doppler was used to verify the identification of the ECA and ICA.

The carotid intima–media thickness was measured when the two echogenic lines, representing the lumen–intima interface and the media–adventitia interface were visualised over a length of ≥ 1 cm. Measurements of the CIMT were done manually, using the calliper markers on the ultrasound unit.

The CIMT at the optimal angle of interrogation was measured bilaterally as the area of maximum thickness at the near and far walls of the CCA, BIF and ICA (a total of 12 sites). In cases where calcified plaque obscured the IMT in the bulb, one wall was measured; the thickest measurement in each segment was imaged and recorded as the final measurement. The mean maximum IMT was recorded as the CIMT. Images were stored on a magnetic optical disc as well as on thermal paper.

The CIMT was calculated as the average of 12 sites for all subjects, using the Excel programme for Windows XP. A mean value for the CIMT of above 0.8 mm was classified as increased thickness.^8^

Subjects with a previous myocardial infarction had to be at least three months post-infarction before recruitment. As the study was designed to assess the effects of cardiovascular risk factors of lifestyle (hypertension, dyslipidaemia, smoking and obesity), subjects were excluded from the study if they were previously diagnosed with diabetes mellitus (DM) or genetic dyslipidaemia, or were positive for HIV infection. Subjects with overt renal, thyroid or liver disease were also excluded. Other exclusions were related to technical considerations which did not allow for measurement of the CIMT, including poor imaging with limited boundary visualisation or when there were anatomical constraints, either a high carotid artery bifurcation or a short, thick neck and where more than two segments were not visualised.

## Statistical analysis

The Excel programme for Windows XP was used to calculate the mean maximum IMT (CIMT) of the carotid arteries. To assess the independent value of the CIMT as a predictor of CAD, linear and multiple regression analyses were done, using the GB–STAT for Windows, version 10, and Dynamic Microsystems Inc 2004.

To find how IMT behaves as a predictor of CAD severity in the presence of other important cardiovascular disease risk factors, we included age, hypertension (> 140/90 mmHg or if the subject was on antihypertension medication), abnormal glucose tolerance, central obesity as assessed by abdominal circumference, dyslipidaemia and cigarette smoking.

## Results

Among the 53 participants, coronary angiography results showed that 15 people had no evidence of CAD. This constituted the control group. Of these, 10 were males and five were females, a ratio of 3 to 1. The mean age was 47 years, with an age range of 30 to 65 years. Although there was no evidence of CAD on angiography, eight of the 15 (53%) subjects had increased CIMT. The mean CIMT was calculated as 0.998 mm. Four subjects had hypertension as the only risk factor for CAD, and the CIMT values of this group had a median CIMT percentile value (1.22 mm) that was as great as that of subjects with multiple risk factors.

The study group consisted of the 38 subjects with evidence of CAD on coronary angiography, defined as lesions greater than 50% in one or more of the major coronary vessels (Table 1). Single-vessel disease was present in 12 patients, double-vessel disease in 11 and triple-vessel disease in 12. Coronary angiography results were not available for three study subjects. In the study population, 29 of the 38 subjects (76%) had increased CIMT, with a mean value of 1.13 mm. The mean age for the study group was 55.4 years, with ages ranging from 36 to 69 years.

**Table 1:** Clinical Characteristics Of The Studied Patients With And Without CAD On Coronary Angiography

	*No CAD (%) (n = 15)*	*CAD (%) (n = 38)*
Hypertension (blood pressure > 140/90 mmHg or on antihypertensive therapy)	60	97
Increased abdominal circumference (male > 92 cm, females > 88 cm)	33.3	79.1
Abnormal glucose tolerance	0	55.26
Raised triglycerides (> 1.7 mmol/l)	26.7	50
Low HDL cholesterol (men < 1.0 mmol/l, women < 1.3 mmol/l)	33.33	42.10
Raised LDL cholesterol (> 3 mmol/l)	13.33	42.10
Cigarette smoking/previous smokers	20	62.5
Mean CIMT (mm)	0.998	1.13
Increased CIMT (> 0.8 mm)	53	76

Linear regression analysis for CIMT against age shows that increasing age was related to increased CIMT (*r* = 0.223, *p* = 0.17). Hypertension, defined as a blood pressure higher than 140/90 mmHg or if the subject was on antihypertensive medication, was very prevalent, 95% of the study subjects being hypertensive. Obesity, particularly abdominal obesity (defined as an abdominal circumference of > 92 cm in males and > 88 cm in females), was also highly prevalent, 79% of the study subjects having increased abdominal circumference. Smoking was also more prevalent in the study group, 62.5 % of the subjects being current or ex-smokers.

Although subjects with diabetes were excluded from the study, 55% were found to have abnormal glucose tolerance. There was a good correlation between CIMT and fasting blood glucose levels of the whole group using simple linear regression analysis (*r* = 0.35, *p* = 0.03). In addition, 100% of the subjects with high fasting blood glucose levels had an increased CIMT.

Of the patients who were found to have impaired glucose metabolism on glucose tolerance tests, 76.2% had increased CIMT. However, those with normal glucose metabolism (neither raised fasting blood glucose nor an abnormal glucose tolerance test) had a similar percentage of subjects with increased CIMT (76.4%). Ninteen patients (5%) had raised fasting triglyceride levels of above 1.7 mmol/l, of which 78.95% had increased CIMT. However the percentage with increased CIMT was similar in the 19 subjects with normal triglyceride levels (73.68%). Subjects with high low-density lipoprotein cholesterol (LDL-C) levels (LDL-C > 3 mmol/l) had increased CIMT in 93.75% of cases. LDL-C levels also showed a strong positive correlation with CIMT.

**Fig. 1.**
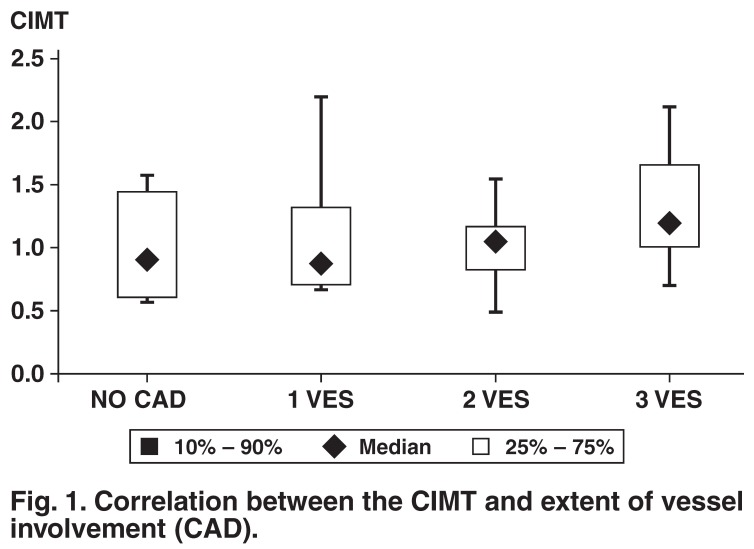
Correlation between the CIMT and extent of vessel involvement (CAD).

The median percentile scores showed a progressive increase as the number of vessels involved increased. The median CIMT average was 0.88 mm in those with single-vessel disease, 1.08 mm in those with double-vessel disease and 1.19 mm in those with triple-vessel disease (Fig. 1). There was a significant positive linear trend between CIMT and the number of vessels involved (*r* = 0.44, *p* < 0.0001).

## Discussion

The study findings support the hypothesis that CIMT is a predictor of the presence and extent of CAD in South African black patients.^9^ Predictors of CIMT included several of the classical CAD risk factors, namely age, hypertension, dyslipidaemia, abdominal obesity, smoking and glucose intolerance. The impact of these risk factors on CIMT has previously been well described.^10^,^11^ On univariate analysis, the key risk factors associated with increased CIMT were hypertension and raised fasting glucose.^12^

Bearing in mind that diabetics were excluded from the study, the effect of high fasting blood glucose levels on the CIMT was significant. In this study, high fasting glucose level, which is also an important component of the metabolic syndrome, was an important factor associated with increased CIMT

The median percentile scores of CIMT showed a progressive increase as the number of coronary vessels involved increased. The findings in the study support previous studies^13^,^14^ where increased CIMT was correlated with evidence of angiographically proven CAD. The study by Kablak-Ziembicka *et al*.^4^ showed that increased CIMT was positively and linearly related to CIMT; subjects with a greater number of vessel involvement showed greater increases in CIMT. In addition, their study showed that a CIMT over 1.15 mm was predictive of a 94% likelihood of having CAD. The study by Geroulakos *et al.*^15^ also showed a significant positive linear trend between CIMT and the number of vessels involved. The study also demonstrated a high positive predictive value and specificity for the presence of CAD if a CIMT above 0.85 mm, correlated for age, is used as a cut-off point for the prediction of CAD.^16^

The limitations are inherent in the study design, which recruited subjects undergoing coronary angiography for a suspected clinical diagnosis of CAD. The study population, a total of 53 subjects, was therefore highly selected, without the possibility of case matching, particularly for the dominant risk factor, hypertension, which was present in every study subject but one.^17^ Future researchers will be faced with the challenge of recruiting a larger group of participants to allow for a controlled study within this population with a rising prevalence of CAD. Better definition of the impact of each of the risk factors on CIMT and consequently CAD should be the role of future studies.
